# Conidial germination in *Scedosporium apiospermum*, *S. aurantiacum*, *S. minutisporum* and *Lomentospora prolificans*: influence of growth conditions and antifungal susceptibility profiles

**DOI:** 10.1590/0074-02760160200

**Published:** 2016-06-27

**Authors:** Thaís Pereira de Mello, Ana Carolina Aor, Simone Santiago Carvalho de Oliveira, Marta Helena Branquinha, André Luis Souza dos Santos

**Affiliations:** Universidade Federal do Rio de Janeiro, Instituto de Microbiologia Paulo de Góes, Departamento de Microbiologia Geral, Laboratório de Investigação de Peptidases, Rio de Janeiro, RJ, Brasil

**Keywords:** Scedosporium, Lomentospora, conidial germination, growth conditions, antifungal susceptibility, morphological changes

## Abstract

In the present study, we have investigated some growth conditions capable of inducing the conidial germination in *Scedosporium apiospermum, S. aurantiacum, S. minutisporum* and *Lomentospora prolificans*. Germination in Sabouraud medium (pH 7.0, 37ºC, 5% CO2) showed to be a typically time-dependent event, reaching ~75% in *S. minutisporum* and > 90% in *S. apiospermum*, *S. aurantiacum* and *L. prolificans after 4 h*. Similar germination rate was observed when conidia were incubated under different media and pHs. Contrarily, temperature and CO2 tension modulated the germination. The isotropic conidial growth (swelling) and germ tube-like projection were evidenced by microscopy and cytometry. Morphometric parameters augmented in a time-dependent fashion, evidencing changes in size and granularity of fungal cells compared with dormant 0 h conidia. In parallel, a clear increase in the mitochondrial activity was measured during the transformation of conidia-into-germinated conidia. Susceptibility profiles to itraconazole, fluconazole, voriconazole, amphotericin B and caspofungin varied regarding each morphotype and each fungal species. Overall, the minimal inhibitory concentrations for hyphae were higher than conidia and germinated conidia, except for caspofungin. Collectively, our study add new data about the conidia-into-hyphae transformation in *Scedosporium* and *Lomentospora* species, which is a relevant biological process of these molds directly connected to their antifungal resistance and pathogenicity mechanisms.

Species belonging to the *Pseudallescheria* and *Scedosporium* genera are saprophytic fungi widely found in human-impacted environments, including soil, water and sediments, which have emerged as etiologic agents of localised and disseminated infections in both immunocompromised and immunocompetent individuals (O’Bryan 2005, Cortez et al. 2008, Kaltseis et al. 2009, Tammer et al. 2011, Kantarcioglu et al. 2012, Lackner & Guarro 2013). Due to the morphological, biochemical and genetic features, some species of *Pseudallescheria* and *Scedosporium* were allocated in a fungal complex designated as *Pseudallescheria/Scedosporium* complex, which is currently composed by *Pseudallescheria boydii*, *Scedosporium apiospermum*, *S. dehoogii*, *S. aurantiacum* and *S. minutisporum* (Gilgado et al. 2010, Lackner et al. 2014). *S. prolificans* (currently *Lomentospora prolificans*) is considered phylogenetically distant from the other species of *Pseudallescheria* and *Scedosporium*; as a consequence, it does not belong to the *Pseudallescheria/Scedosporium* complex (Lackner et al. 2014).

*Scedosporium* species are the second most frequently isolated fungi, just after *Aspergillus fumigatus,* recovered from patients with cystic fibrosis, which is characterised by defective mucociliary clearance that provides an ideal environment for the full development of airborne conidia in the lung of individuals carrying this genetically inherited disorder (Blyth et al. 2010, Lackner et al. 2012). Classically, *Scedosporium* spp. are mainly associated with white-grain mycetoma and subcutaneous infections in cartilage and joint areas, in which the most affected population are immunologically healthy individuals, who suffer traumatic inoculation of conidial cells and/or mycelial fragments. However, in recent years a growing number of invasive and disseminated infections have been reported (Cortez et al. 2008, Kaltseis et al. 2009, Lackner & Guarro 2013). Invasive cases caused by *Scedosporium* usually start with inhalation of airborne conidia followed by their adhesion to the lung tissue. Subsequently, conidial cells differentiate into hyphae inside the respiratory tract of individuals with predisposing conditions such as advanced human immunodeficiency virus (HIV) infection, chronic granulomatous disease, hematological malignancies, transplantation recipients and near-drowning accident victims (O’Bryan 2005, Cortez et al. 2008, Tintelnot et al. 2008, Kaltseis et al. 2009, Tammer et al. 2011, Kantarcioglu et al. 2012, Lackner & Guarro 2013).

Fungal germination comprises the processes and changes occurring during the resumption of development of a resting cell and its transformation to a morphologically different structure, which involves the conversion from a nonpolar cell into a polar germ tube-like projection, growing by extension at the tip. Three stages in the process may be visually distinguished: (i) a preliminary stage of swelling (isotropic growth), (ii) the establishment of polarisation and the emergence of the germ tube-like projection, and (iii) the full hyphal development (Allen 1965, D’Enfert 1997, Osherov & May 2001). The morphological transition of conidia into hyphae is a critical step during the life cycle and pathogenesis of filamentous fungi (van Burik & Magee 2001, Gow et al. 2002) and throughout this process several morphophysiological changes occur in the fungal cells (Wächtler et al. 2012, Gilmore et al. 2013). For example, in dormant conidia of *A. fumigatus*, the inner cell wall components (e.g., chitin and β-glucan) are masked by an inert hydrophobic rod let layer that is degraded upon swelling and germination steps, exposing the underlying carbohydrate layers (Dague et al. 2008, Aimanianda et al. 2009). In *S. apiospermum*, ceramide monohexoside (CMH) was found at the surface of mycelia, but it was not detected at the surface of conidial cells by means of immunofluorescence microscopy analysis using anti-CMH antibody (Pinto et al. 2002). The modulation on the expression/exposition of surface-located molecules (i) culminates in different ability to adhere on both abiotic and biotic surfaces, (ii) promotes the escape from host immune responses and (iii) induces changes concerning the susceptibility to antifungal drugs (Osherov & May 2001). Differences in minimum inhibitory concentration (MIC) values for filamentous fungi were reported when conidia (fungus in the lag phase) and hyphae (fungus in the log or stationary growth phase) were tested (Guarro et al. 1997, Manavathu et al. 1999, Meletiadis et al. 2001, Osherov & May 2001, van de Sande et al. 2010, Lackner et al. 2012).

As a complex and multifaceted event, cellular differentiation is finely orchestrated and controlled at different cellular levels (D’Enfert 1997, Osherov & May 2001). It is well-known that some environmental conditions such as pH, temperature, nutrient availability, oxygen and carbon dioxide (CO_2_) are potent inducers of the differentiation process in fungi (Wächtler et al. 2012, Gilmore et al. 2013). In order to add new data on this relevant subject, in the present study we have investigated some physicochemical conditions able to induce the conidial germination in *S. apiospermum*, *S. aurantiacum*, *S. minutisporum* and *L. prolificans*, including culture medium composition, pH, temperature and CO_2_ tension. In addition, we have compared the susceptibility profile of conidia, germinated conidia and hyphae of these human opportunistic filamentous fungi to classical antifungal drugs (itraconazole, fluconazole, voriconazole, caspofungin and amphotericin B).

## MATERIALS AND METHODS

*Microorganisms and growth conditions - S. apiospermum* (strain HLBP) was provided by Dr Bodo Wanke (Hospital Evandro Chagas, Instituto Oswaldo Cruz, Rio de Janeiro, Brazil), *L. prolificans* (strain FMR 3569) was provided by Dr Josep Guarro (Facultad de Medicina y Ciencias de la Salud, Reus, Spain), *S. minutisporum* (strain IHEM21148) and *S. aurantiacum* (strain IHEM21147) were provided by Dr Jean-Philippe Bouchara (Université d’Angers, Angers, France). The fungi were maintained on Sabouraud (2% glucose, 1% peptone, 0.5% yeast extract, pH 7.0) liquid culture medium for seven days at room temperature with orbital shaking (200 rpm) (Pinto et al. 2002, 2004, Silva et al. 2006). To obtain the conidial cells, each fungus was grown at room temperature on Petri dishes containing potato dextrose agar (PDA; Difco Laboratories, USA). After seven days in culture, conidia were obtained by washing the plate surface with phosphate-buffered saline (PBS; 10 mM NaH_2_PO_4_, 10 mM Na_2_HPO_4_, 150 mM NaCl, pH 7.2) and filtering them through a 40-µm nylon cell strainer (BD Falcon, USA) in order to remove the hyphal fragments (Hohl et al. 2005, Silva et al. 2006). The conidial cells were counted in a Neubauer chamber.

*Conidial germination assay* - Conidial suspension (5 × 10^5^ cells/µL, total volume of 20 µL) was transferred into each well of a 96-well polystyrene microtiter plates (Corning^®^, Corning Incorporated, USA) containing 180 µL of Sabouraud medium (pH 7.0), up to 4 h at 37ºC with 5% CO_2_. After each time point (1, 2, 3 and 4 h), the number of conidia and germinated conidia were counted in an inverted microscope (Zeiss, Germany). At least 200 fungal cells were counted per well in each system (Silva et al. 2011) and the results were expressed as percentage of germinated conidia in comparison to remaining conidial cells. In parallel, the fungal viability was assessed by the colorimetric assay that investigates the metabolic reduction of 2,3-bis (2-methoxy-4-nitro-5-sulfophenyl)-5-[(phenylamino) carbonyl]-2H-tetrazolium hydroxide (XTT; Sigma-Aldrich, St. Louis, MO, USA) to a water-soluble brown formazan product in mitochondria (Mowat et al. 2007, Peeters et al. 2008). In this sense, 100 µL of the XTT/menadione solution [4 mg XTT dissolved in 10 mL pre warmed PBS and supplemented by 100 µL menadione stock solution (Sigma-Aldrich, St. Louis, MO, USA), which contained 55 mg menadione in 100 mL acetone] was added to all wells and incubated in the dark at 37ºC for 4 h. The contents of the wells were transferred to micro centrifuge tubes and centrifuged at 4,000×g for 5 min. A total of 100 µL of the supernatant from each well was transferred to a new microplate and the colorimetric changes were measured at 492 nm using a microplate reader (SpectraMax M3; Molecular Devices, USA).

**Fig. 1:**
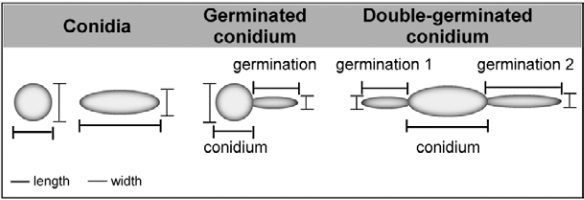
: representative drawings of conidia and germinated conidia observed in species of *Scedosporium* and *Lomentospora*. Two distinct morphological dimensions (length and width) can be evaluated in each conidium, germinated conidium and germ tube-like projection (germination), as shown in the scheme.

*Morphological parameters* - Two morphological parameters (size and granularity) were evaluated along the conidial germination of *S. apiospermum*, *S. aurantiacum*, *S. minutisporum* and *L. prolificans* by means of flow cytometry (BD FACSCalibur, BD Biosciences, USA) (Santos et al. 2012, Hayer et al. 2013). The germination assay was carried out as described previously. After 1, 2, 3 and 4 h, we observed that the conidia and germinated conidia were able to adhere to the wells of the polystyrene micro centrifuge tubes. In order to detach the fungal cells, the systems were centrifuged at 4,000×g for 5 min to remove Sabouraud medium and added of a solution (1 mL) containing trypsin-ethylenediaminetetraacetic acid (0.25% trypsin and 1 mM EDTA; Sigma-Aldrich, St. Louis, MO, USA) for 5 min at 37ºC. Then, the micro centrifuge tubes were harvested by centrifugation to remove the trypsin and added of a solution (1 mL) containing 0.01% Tween 80 (Sigma-Aldrich, St. Louis, MO, USA). The systems were vortexed for 1 min to release the adhered fungal cells. The cells were washed once with cold PBS and fixed in 4% paraformaldehyde at 4ºC for 30 min. Each fungal population was mapped (30,000 events) using a two-parameter histogram of forward-angle light scatter (FSC) *versus* side scatter (SSC), in order to evaluate size and granularity, respectively (Santos et al. 2012, Hayer et al. 2013). In parallel, the morphology of *S. apiospermum*, *S. aurantiacum*, *S. minutisporum* and *L. prolificans* cells were evaluated under light microscopy using a Zeiss Axioplan 2 microscope with a 63´ objective lens and a final magnification of ´630 (Zeiss, Germany) (Silva et al. 2011). The dimensions (length and width) of 50 conidia and 50 germinated conidia of each species were measured as exemplified in Fig. 1. Considering the germinated conidia, the length and width of both conidium body and germination projection were separately measured (Fig. 1). In the specific case of double-germinated conidia (Fig. 1), the final length and width of the germination projections were considered as the arithmetic mean of the individual measurements considering each analysed parameter.

*Modulation of conidial germination by physicochemical conditions* - In this set of experiments, the fungal germination was evaluated by incubating the conidial cells under different growth conditions. In this way, conidia (10^4^ cells) were incubated for 4 h in distinct (i) culture media [Dulbecco’s modified Eagle’s medium (DMEM; Sigma-Aldrich, St. Louis, MO, USA), fetal bovine serum (FBS; Gibco, Life Technologies, USA) and Sabouraud], (ii) pH values (5.0, 7.0 and 9.0), temperatures (21ºC, 37ºC and 40ºC) and CO_2_ tensions (0.033% and 5%). The number of fungal morphotypes and viability were analysed as described above.

*In vitro susceptibility testing* - The in vitro antifungal susceptibility testing was performed using different fungal morphotypes (initial inoculum of 10^4^ fungal cells). Conidia, 4 h-old-germinated conidia and 16 h-old-germinated conidia (in which just mycelia were observed) were used to investigate their susceptibility profiles to itraconazole (concentrations ranging from 0.03-128 µg/mL), fluconazole (0.03-256 µg/mL), voriconazole (0.03-128 µg/mL), caspofungin (0.06-128 µg/mL) and amphotericin B (0.03-128 µg/mL) (Sigma-Aldrich, St. Louis, MO, USA) by using broth microdilution method standardised for conidial cells of filamentous fungi according to the document M38-A2 published by the Clinical and Laboratory Standards Institute (CLSI 2008) and for hyphal cells as earlier proposed by Bezjak (1985). The plates were then incubated for 48 h at 37ºC. The MICs were determined by visual inspection and confirmed with XTT-based reduction assay as the least concentration with no XTT reduction, which characterises the fungal cells with inactive metabolism. As recommended by CLSI, *Candida krusei* (ATCC 6258) and *C. parapsilosis* (ATCC 22019) were used as quality control isolates in each test. In all the susceptibility experiments, systems containing Roswell Park Memorial Institute (RPMI, Sigma-Aldrich, St. Louis, MO, USA) medium plus fungal suspension, RPMI plus tested antifungal drugs solutions (dissolved in dimethylsulfoxide - DMSO, Sigma-Aldrich, St. Louis, MO, USA), RPMI plus DMSO, RPMI plus DMSO plus fungal suspension and RPMI only were used as controls.

*Statistics* - All the experiments were performed in triplicate, in three independent experimental sets. The data were expressed as mean ± standard deviation. Results were evaluated by Student’s *t*-test using Graphpad Prism 5 computer software. In all analyses, *p* values of 0.05 or less were considered statistically significant.

## RESULTS AND DISCUSSION

*Time-dependence of conidial germination* - Germination is a key event in fungal pathogenesis, because it allows the pathogen to be capable of adhering, spreading and invading different cells and tissues in the host (D’Enfert 1997, Osherov & May 2001, van Burik & Magee 2001, Gow et al. 2002). For instance, the susceptibility of the insect larvae of *Galleria mellonella* to infection by *A. fumigatus* was directly dependent upon the stage of conidial germination, as follows: non-germinating (or resting) conidia < early stages of the germination < outgrowth phase of germination (Renwick et al. 2006). Furthermore, the examination of the immune response of *G. mellonella* to the fungal infection revealed that hemocytes were able to engulf non-germinating conidia and those in the early stages of the germination process, while conidia that reached the outgrowth stages of germination were not phagocytosed (Renwick et al. 2006). However, the mechanisms underlying this essential process remain poorly understood in filamentous fungi, especially in species belonging to the *Scedosporium/Pseudallescheria* complex as well as related species like *L. prolificans*.

**Fig. 2:**
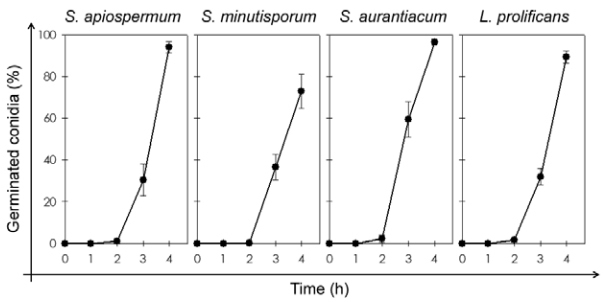
: time-dependence kinetics of conidial germination in *Scedosporium apiospermum*, *S. minutisporum*, *S. aurantiacum* and *Lomentospora prolificans*. Conidial cells were incubated in Sabouraud medium at 37ºC in an atmosphere of 5% CO2. After 0, 1, 2, 3 and 4 h of incubation, the number of non-germinated and germinated conidial cells (please, see representative images in Fig. 1) were counted by using an inverted microscope. The results are expressed as percentage of germinated conidia in comparison to remaining conidial cells. The results are shown as the mean ± standard deviation of three independent experiments.

The term germination usually implies the emergence of a definite germ tube-like projection from conidial cell (Allen 1965, D’Enfert 1997). Taking it into consideration, we initially analysed the time-dependence kinetics of the morphological transformation after incubation of *S. apiospermum*, *S. aurantiacum*, *S. minutisporum* and *L. prolificans* conidial cells in Sabouraud medium (pH 7.0) at 37ºC up to 4 h in an atmosphere of 5% CO_2_ (Fig. 2). No conidial germination was detected in the first hour of incubation under the employed experimental conditions, while an extremely low germination rate (< 3%) was observed in the studied fungi after 2 h (Fig. 2). These rates significantly increased after 3 h of incubation to 30.5%, 59.4%, 36.6% and 32.0% in *S. apiospermum*, *S. aurantiacum*, *S. minutisporum* and *L. prolificans,* respectively. After 4 h, the germination reached around 75% in *S. minutisporum* and more than 90% in *S. apiospermum, S. aurantiacum* and *L. prolificans* (Fig. 2).

Meletiadis et al. (2001) showed that the germination of *L. prolificans* conidial cells started only after 4 h of incubation in Sabouraud and antibiotic medium 3 (AM3), after 5 h of incubation in RPMI and RPMI supplemented with 2% glucose and after 7 h of incubation in yeast nitrogen base (YNB). In addition, complete germination was not achieved in any of the tested media even after 20 h of incubation at 37ºC in atmospheric concentration of CO_2_ (0.033%). In *A. fumigatus*, the germination of conidia started after 5 h of incubation in the five nutrient media (AM3, YNB, Sabouraud, RPMI alone and RPMI with 2% glucose), although it was delayed by 1.5 h in YNB medium (Meletiadis et al. 2001). Ghamrawi et al. (2015) found around 30% of germinated conidia in *S. boydii* after incubation in yeast peptone dextrose for 4 h at 37ºC in atmospheric concentration of CO_2_. All these controversial results, including our own findings, could be explained due to the employment of different growth conditions such as culture medium composition and CO_2_ concentration, which are two relevant parameters that modulate the differentiation process in several fungi (Allen 1965, D’Enfert 1997, Osherov & May 2001, Wächtler et al. 2012, Gilmore et al. 2013).

**Fig. 3:**
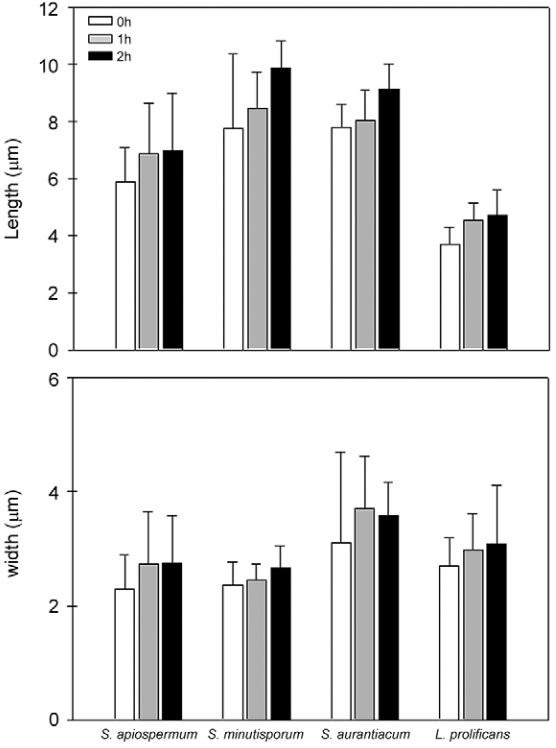
: morphological dimensions evaluated under light microscopy of the pre-germinative stages from conidial cells of *Scedosporium apiospermum*, *S. minutisporum*, *S. aurantiacum* and *Lomentospora prolificans*. In this set of experiments, conidial cells were incubated in Sabouraud medium at 37ºC in an atmosphere of 5% CO2 up to 2 h. After 0, 1 and 2 h, the length and width of 50 conidia were measured. The results are shown as the mean ± standard deviation of three independent experiments.

An early visual indicator of conidial germination involves the isotropic swelling of conidia before switching to polarised growth, which results in the formation of a germ tube-like emergence and further mycelial development (Allen 1965, D’Enfert 1997). The results summarised in Fig. 2 suggested that in the first 2 h of incubation, under the employed conditions in the present study, *S. apiospermum*, *S. aurantiacum*, *S. minutisporum* and *L. prolificans* conidial cells did not develop the germ tube extension, probably because it was the period of conidial isotropic growth. In order to verify this hypothesis, the length and width of conidial cells were measured after 0, 1 and 2 h (Fig. 3). Our results showed that the conidial length increased around 15-30% and the conidial width around 15-20% after 2 h (Fig. 3). However, no significant differences on both morphological parameters (length and width) were observed for any of the studied fungi at this time interval (Fig. 3). In contrast, during the conidial swelling of *A. niger*, *Fusarium oxysporum*, *Penicillium discolor* and *Verticillium fungicola*, the diameter of the conidia increased two-fold or more and it involved water uptake and a decrease in the micro-viscosity of the cytoplasm (van Leeuwen et al. 2010).

**Fig. 4:**
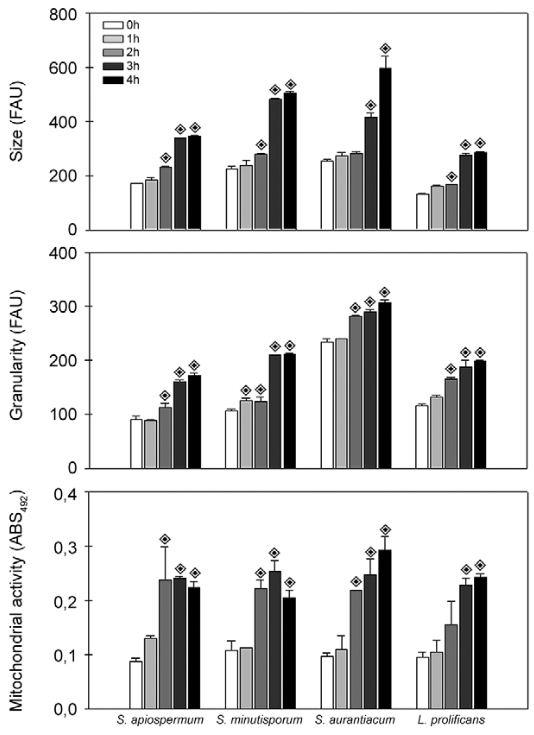
: morphological parameters and metabolic activity evaluated during the transformation from conidia to germinated conidia of *Scedosporium apiospermum*, *S. minutisporum*, *S. aurantiacum* and *Lomentospora prolificans*. In this set of experiments, conidial cells were incubated in Sabouraud medium at 37ºC in an atmosphere of 5% CO2 up to 4 h. After 0, 1, 2, 3 and 4 h, fungal cells were processed to estimate the size (forward scatter parameter) and granularity (side scatter parameter) by flow cytometry analysis and the results were expressed as fluorescence arbitrary units (FAU). In parallel, the mitochondrial activity was measured by monitoring the metabolic reduction of XTT at 492 nm using a microplate reader. The results are shown as the mean ± standard deviation of three independent experiments. *p* values were obtained comparing the dormant 0 h conidial cells to the other time-points in which the conidia were collected, and the diamond symbols indicate that *p* < 0.05 (Student’s *t* test).

Germination process was also monitored using flow cytometry through modifications in cell size (FSC) and granularity (SSC) as previously reported by *A. niger*, in which the first parameter provides quantifiable data on conidial swelling (Hayer et al. 2013). Corroborating the data exposed in Fig. 3, the FACS analysis revealed an augmentation around 15-40% in the conidia size during the pre-germinative stage (comparison between 0-2 h) (Fig. 4). However, in view of the whole germination process (4 h), our results revealed that both morphometric parameters increased in a time-dependent manner (Fig. 4A-B), corroborating changes on the size and granularity (internal complexity) of fungal cells compared to dormant 0-h conidia. These results are in accordance with biological processes required for conidial germination such as increase in metabolic activities, including synthesis of new RNA, proteins and molecules that constitute the new membranes and cell wall being formed (Osherov & May 2001). Further on, changes in the expression of surface molecules and in the cellular architecture were reported. For example, the surface of *A. fumigatus* conidial cells contain hydrophobins and melanin, while germinated conidia presented a-1,3-glucan, galactomannan and galactosaminogalactan exposed at the cell wall (Latgé & Beauvais 2014). The lipid composition of the plasma membrane also changes by the appearance of sterol-rich domains (van Leeuwen et al. 2010). Moreover, it is well-known that there is a rise in both endogenous respiratory rate and the rate of oxidation of carbon sources under conditions that permit germination. Conversely, if the external conditions needed for germination are removed, the rise in respiratory potential stops (Allen 1965, Osherov & May 2001). Supporting this statement, *A. fumigatus* was unable to germinate under anaerobic conditions; contrarily, active mitochondria were evidenced by fluorescent mitotracker dye already at the stage of swollen conidia, which indicated that respiration is an early event during germination (Taubitz et al. 2007). In this sense, a clear time-dependent augmentation in the mitochondrial activity, as determined by the metabolic reduction of XTT, was evidenced during the transformation of conidia into germinated conidia of *S. apiospermum*, *S. aurantiacum*, *S. minutisporum* and *L. prolificans* (Fig. 4C).

**Fig. 5:**
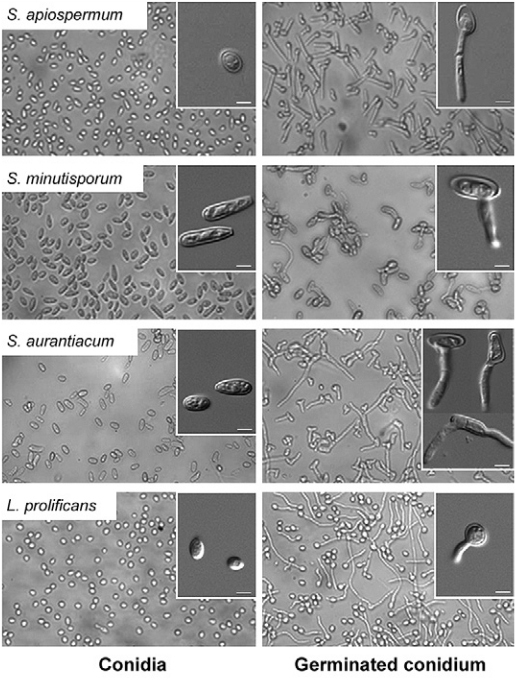
: light microscopies showing the dormant (0 h-conidia) and germinated conidial cells of *Scedosporium apiospermum*, *S. minutisporum*, *S. aurantiacum* and *Lomentospora prolificans* after 4 h of incubation in Sabouraud medium at 37ºC in an atmosphere of 5% CO2. The insets highlighted the different morphologies regarding both conidia and germinated conidia observed in each studied fungus. Bars represent 4 μm.

Subsequently, we performed an inspection of both conidial and germinated conidial cells by light microscopy in order to better visualise these different fungal morphotypes (Fig. 5). The conidia of *S. apiospermum* were ovals in shape, measuring approximately 5.9 ± 1.2 µm x 2.3 ± 0.6 µm, and the germination projection was observed only from one site (pole) of each conidium, measuring around 11.2 ± 4.0 µm x 0.2 ± 0.1 µm (Fig. 5, Table I). The conidia of *S. minutisporum* had ellipsoidal shape, with one of the straight edges and the other one rounded, measuring 7.8 ± 2.6 µm x 2.4 ± 0.4 µm and the germination was observed emerging from the central part of the conidial cell, measuring around 7.0 ± 1.6 µm x 0.2 ± 0.1 µm (Fig. 5, Table I). The conidia of *S. aurantiacum* were oval to cylindrical, measuring about 7.8 ± 0.8 µm x 3.1 ± 1.6 µm and the germination (11.2 ± 2.6 µm x 0.2 ± 0.1) can emerge from the middle, from one extremity or from both conidial tips (Fig. 5, Table I). Germination in both ends is called “bipolar germ-cell” and was also observed in other fungal species, such as *Ashbya gossypii* (Wendland & Philippsen 2001). Interestingly, this kind of morphology allows a more efficiently space exploration (Harris 2005). The conidia of *L. prolificans* had oval or globose shapes, measuring 3.7 ± 0.6 µm x 2.7 ± 0.5 µm, and the germination projection (7.8 ± 3.1 µm x 0.2 ± 0.1 µm) can appear in any part of the conidial surface (Fig. 5, Table I).

**TABLE I:** Length and width of conidia and germinated conidia of *Scedosporium apiospermum*, *S. minutisporum*, *S. aurantiacum* and *Lomentospora prolificans*

Cell dimensions (µm)	*S. apiospermum*			*S. minutisporum*			*S. aurantiacum*			*L. prolificans*		
			
	Conidia	germinated conidia		conidia	germinated conidia		conidia	germinated conidia		conidia	germinated conidia	
		conidia	germination		conidia	germinated		conidia	germinated		conidia	germinated
Length	5.9 ± 1.2	5.7 ± 1.8	11.2 ± 4.0	7.8 ± 2.6	8.6 ± 1.3	7.0 ± 1.6	7.8 ± 0.8	7.1 ± 1.4	11.2 ± 2.6	3.7 ± 0.6	3.9 ± 0.6	7.8 ± 3.1
Width	2.3 ± 0.6	2.1 ± 0.8	0.2 ± 0.1	2.4 ± 0.4	2.4 ± 0.8	0.2 ± 0.1	3.1 ± 1.6	3.1 ± 0.6	0.2 ± 0.1	2.7 ± 0.5	4.3 ± 1.0	0.2 ± 0.1

*Effect of culture medium and pH on conidial germination* - It is well-known that conidial germination occurs in environments containing available water and the appropriate concentration of low molecular mass nutrients, like sugars, amino acids and inorganic acids (Osherov & May 2001). For example, water supplemented with D-glucose was sufficient to enable conidial germination of *A. niger* (Hayer et al. 2013) *. A. fumigatus* germination and hyphal growth in the mammalian lung, following the survival of resident pulmonary defenses, require the activation of nutrient-sensing, acquisition and biosynthetic pathways to obtain nutrients from the host environment (Dagenais & Keller 2009).

**Fig. 6:**
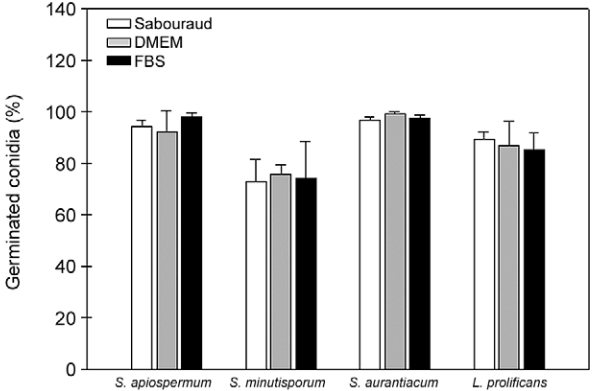
: evaluation of conidial germination of *Scedosporium apiospermum*, *S. minutisporum*, *S. aurantiacum* and *Lomentospora prolificans* in different culture media. Conidial cells were incubated for 4 h in Sabouraud, Dulbecco’s modified Eagle’s medium (DMEM) or fetal bovine serum (FBS) at 37ºC with an atmosphere of 5% CO2. After incubation, fungal cells were counted by using an inverted microscope. The results are shown as the mean ± standard deviation of three independent experiments. There was no statistical difference in the germination of each fungal species when cultured under these different culture media.

In order to evaluate the conidial germination of *S. apiospermum*, *S. aurantiacum*, *S. minutisporum* and *L. prolificans* under cultivation in different growth media, conidia were incubated in Sabouraud, DMEM and FBS at neutral pH at 37ºC for 4 h in an atmosphere of 5% CO_2_. In all fungal species, no significant difference regarding the germination rate was observed among the culture media studied; however, *S. minutisporum* presented a lower rate of differentiation (74.3%) compared to *S. apiospermum* (94.9%), *S. aurantiacum* (97.8%) and *L. prolificans* (87.2%) (Fig. 6). Sabouraud medium was selected for the further experiments because it is the culture medium used to the growth of all these fungi.

**Fig. 7:**
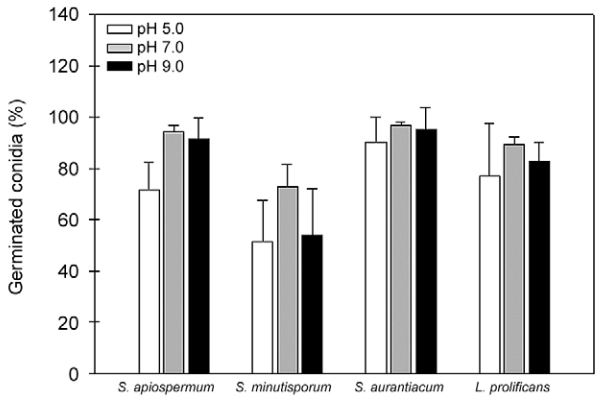
: evaluation of conidial germination of *Scedosporium apiospermum*, *S. minutisporum*, *S. aurantiacum* and *Lomentospora prolificans* in different pH values. Conidial cells were incubated for 4 h at 37ºC with an atmosphere of 5% CO2 in Sabouraud medium in which the pH was adjusted to 5.0, 7.0 and 9.0. After incubation, fungal cells were counted by using an inverted microscope. The results are shown as the mean ± standard deviation of three independent experiments. There was no statistical difference in the germination of each fungal species when cultured in different pH values.

Subsequently, the influence of pH on the germination process was studied by incubating conidial cells in Sabouraud medium buffered at acidic, neutral and alkaline pH for 4 h at 37ºC with 5% CO_2_. It is important to highlight that fungal viability was not affected by the incubation under different pH values (data not shown). Conidia of each fungal species were able to germinate in very similar rates regardless of pH value (Fig. 7). We believe that pH did not influence the conidial germination because all the nutrients required to this process were satisfactorily available in the studied media (Carlile et al. 2001). Once again, *S. minutisporum* showed the lowest percentage of germination compared to the other studied fungal species concerning each analysed pH (Fig. 7). Several fungal species are also capable of differentiating in a broad range of pH; as an example, *Potebniamyces pyri* conidial cells were able to fully germinate in pH values ranging from 4.0-7.0 (Liu & Xiao 2005).

Collectively, these results demonstrated the ability of *S. apiospermum*, *S. aurantiacum*, *S. minutisporum* and *L. prolificans* conidia to differentiate at acid-to-basic environments as well as in different nutritional media, which can reflect their prodigious ability to colonise and to invade different sites of human body and natural environments (Cortez et al. 2008, Kaltseis et al. 2009).

*Effect of temperature and CO*_*2*_ *on conidial germination* - The ability of a microorganism to grow in human body temperature under both normal and fever conditions is an important requisite to cause systemic infection (van Burik & Magee 2001). Another interesting parameter to be analysed is the CO_2_ level. In mammalian tissues, the concentration of CO_2_ is approximately 150-fold higher when compared to the atmospheric CO_2_ concentration; consequently, pathogens are exposed to drastic differences considering superficial infections in comparison to deep infections (Klengel et al. 2005). Herein, it was evaluated whether the conidia of *S. apiospermum*, *S. aurantiacum*, *S. minutisporum* and *L. prolificans* were able to differentiate under temperature conditions simulating environmental (21ºC), healthy human body (37ºC) and fever condition (40ºC) in the presence of 0.033% (atmospheric level) or 5% (concentration found in mammalian tissues) of CO_2_.

**Fig. 8:**
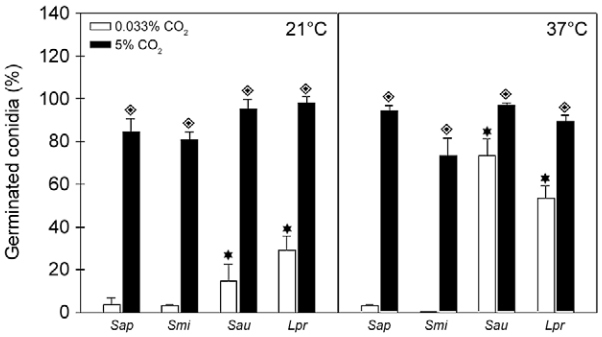
: evaluation of conidial germination of *Scedosporium apiospermum* (*Sap*), *S. minutisporum* (*Smi*), *S. aurantiacum* (*Sau*) and *Lomentospora prolificans* (*Lpr*) in different temperatures and CO2 tensions. Conidial cells were incubated for 4 h on Sabouraud medium at 21ºC and 37ºC in an atmosphere of 5% CO2 or 0.033% CO2. After incubation, fungal cells were counted by using an inverted microscope. The results are shown as the mean ± standard deviation of three independent experiments. Diamonds represent the significant difference (*p* < 0.05, Student’s *t* test) when the germination of each fungus was compared under different CO2 tensions (5% or 0.033% CO2), while stars represent the significant difference (*p* < 0.05, Student’s *t* test) when the germination of each fungus was compared under different temperatures (21ºC and 37ºC).

Firstly, we analysed the differentiation of conidia at temperatures of 21ºC, 37ºC and 40ºC in an environment containing 5% CO_2_, and the results demonstrated a comparable ability of conidial cells to differentiate after 4 h of incubation either at 21ºC or 37ºC (Fig. 8); however, germination was not detected at 40ºC in any studied fungi (data not shown). Furthermore, conidia of *S. apiospermum* and *S. minutisporum* did not differentiate even after incubation for 16 h at 40ºC in an environment with 5% of CO_2_; however, conidia of *S. aurantiacum* and *L. prolificans* were able to fully germinate under these conditions (data not shown). Secondly, a completely distinct profile was observed when the studied fungi were incubated in a lower concentration of CO_2_. In this sense, after 4 h of incubation at 21ºC under atmospheric concentration of CO_2_, only 3.9% of *S. apiospermum*, 14.8% of *S. aurantiacum*, 3.5% of *S. minutisporum* and 29.2% of *L. prolificans* conidia were capable of germinating (Fig. 8). When the temperature was changed to 37ºC (0.033% CO_2_), a considerable increase in the germination rate of *S. aurantiacum* (73.3%) and *L. prolificans* (53.5%) was detected, while no alteration regarding the germination level was observed in *S. apiospermum* and *S. minutisporum* (Fig. 8). Finally, the fungi studied herein were not able to differentiate after incubation for 4 h at 40ºC with atmospheric concentration of CO_2_. Due to this later result, we decided to conduct an additional set of experiment in order to evaluate the conidial viability. By checking their mitochondrial activity, all the fungal species analysed presented similar ability to convert XTT in formazan after incubation under the atmospheric concentration of CO_2_ for 4 h at 21ºC, 37ºC and 40ºC, being all these conditions able to sustain the fungal viability (data not shown). Kaur et al. (2015) demonstrated that both clinical and environmental strains of *S. aurantiacum* presenting higher level of virulence also displayed flexibility and metabolic adaptability to different temperatures ranging from 28ºC to 37ºC. Several studies suggested that the germination rate of *Aspergillus* spp. at 37ºC correlate with pathogenicity in multiple animal models of invasive aspergillosis (Dagenais & Keller 2009). The germination rates of *A. fumigatus*, *A. flavus* and *A. niger* were similar at temperatures up to 30ºC, but differed at 37ºC and 42ºC (Araujo & Rodrigues 2004).

CO_2_ is long known to be an essential factor for the germination of bacterial spores. Similarly, in *Aspergillus* conidia, CO_2_ is one of the essential factors for the initiation of germination (Yanagita 1957). Yanagita (1963) found that, after the germination of *A. niger* conidia had started, the incorporation of ^14^CO_2_ proceeded actively without any lag, and macromolecular substances, such as nucleic acids and proteins, were labeled very rapidly. The importance of 5% CO_2_ during the conidial germination process in *S. apiospermum*, *S. aurantiacum*, *S. boydii*, *S. minutisporum* and *L. prolificans* is corroborated by studies done by other authors, who analysed conidial germination in *L. prolificans* (Meletiadis et al. 2001), *S. apiospermum* and *S. boydii* (Pinto et al. 2004, Santos et al. 2009, Lopes et al. 2010, Ghamrawi et al. 2015) after incubation at both room temperature and 37ºC under environmental atmospheric concentration of CO_2_. In all those works, the authors found low percentages of germinated conidia.

CO_2_ levels can change the physiology of fungal cells through changes in environmental acidity and those levels are used by fungal pathogens as a signal for modulating the expression of virulence factors (Lang-Yona et al. 2013). For example, an atmosphere of 5% CO_2_ induced the pseudohyphae formation in *C. albicans* as well as capsule production in *Cryptococcus neoformans* (Klengel et al. 2005, Mitchell 2005). Conidia of *Aspergillus* were not able to germinate in the absence of CO_2_, even when cultivated in medium containing all the essential nutrients that allow full growth (Yanagita 1957). These results showed that the germination of conidia was highly induced by the presence of CO_2_ and thus favor differentiation in environments with this condition, such as mammalian tissues and bloodstream.

*Antifungal susceptibility of conidia, germinated conidia and hyphae* - Traditionally, standardised methods for in vitro susceptibility testing of filamentous fungi use exclusively inoculum of conidia, which are not the unique and/or prevalent morphological form found in the tissues/organs of the infected host. In this context, most infections caused by filamentous fungi are characterised by the presence of hyphal elements in tissue (van Burik & Magee 2001, Gow et al. 2002, Araujo & Rodrigues 2004). Tests performed with hyphae could then predict the therapeutic potential of a drug, which could avoid treatment failures (van de Sande et al. 2010). Corroborating these findings, conidial and mycelial cells are usually found in human tissues infected by *Scedosporium* species (Cortez et al. 2008). Despite these data, almost nothing is known about the influence of different morphotypes on the susceptibility profiles to current antifungal drugs (Wetter et al. 2005). Aggravating this scenario, the few published studies comparing the susceptibility of conidia and hyphae of filamentous fungi are controversial. For instance, the results published in the literature with conidial and hyphal forms of *A. fumigatus* revealed that the MIC values were similar for amphotericin B, itraconazole, voriconazole and posaconazole (Bezjak 1985, Manavathu et al. 1999, Wetter et al. 2005). On the other hand, experiments conducted with species belonging to the *Scopulariopsis, Paecilomyces, Cladosporium* and *Cladophialophora* genera demonstrated that MICs for hyphae were higher than for conidia when the antifungals amphotericin B, fluconazole, ketoconazole, flucytosine, miconazole and itraconazole were employed (Guarro et al. 1997).

In this work, we performed the susceptibility test in order to identify possible differences among conidia, germinated conidia and hyphae of *S. apiospermum*, *S. aurantiacum*, *S. minutisporum* and *L. prolificans*. Our results showed that the antifungal susceptibility profiles varied regarding each morphotype and each fungal species (Table II). In general, the MICs for hyphae were practically always substantially higher than for conidia and germinated conidia. The exception to this profile was caspofungin, for which the MIC values for hyphae were lower than for the remaining morphotypes in all tested fungi (Table II). Although in this work only one strain of each fungal species was studied, the MIC values found herein are in complete agreement with the MICs published by other authors, who worked with several strains (Gilgado et al. 2006, Wiederhold & Lewis 2009, Lackner et al. 2012). In this context, our data (Table II) confirm previously published results regarding the high degree of multidrug resistance of *L. prolificans* to antifungals irrespective of the method of detection used (Alvarez et al. 1995, Lackner et al. 2012), whereas *S. minutisporum* was the more susceptible species to azoles (Lackner et al. 2012).

**TABLE II:** Susceptibility profiles (minimal inhibiotry concentration in µg/mL) of conidia, germinated conidia and hyphae of *Scedosporium apiospermum*, *S. minutisporum*, *S. aurantiacum* and *Lomentospora prolificans* to different antifungals

Antifungal drugs (range in µg/mL)	*S. apiospermum*			*S. minutisporum*			*S. aurantiacum*			*L. prolificans*		
			
	conidia	germinated conidia	hyphae	conidia	germinated conidia	hyphae	conidia	germinated conidia	hyphae	conidia	germinated conidia	hyphae
Itraconazole (0.03-128)	8	8	128	0.5	0.5	32	4	1	128	64	> 128	> 128
Fluconazole (0.125-256)	16	> 256	> 256	0.03	4	256	4	4	> 256	> 256	> 256	> 256
Voriconazole (0.03-128)	0.125	0.5	16	0.25	0.125	32	0.125	0.125	128	8	4	8
Caspofungin (0.06-128)	64	32	8	32	32	16	64	64	32	64	32	8
Amphotericin B (0.06-128)	> 128	> 128	> 128	> 128	> 128	> 128	> 128	> 128	> 128	> 128	> 128	> 128

Despite the increasing number of cases of infections caused by *Pseudallescheria*/*Scedosporium* and *Lomentospora* species, there are no validated interpretive breakpoints for determining resistance to clinically used antifungals (Cortez et al. 2008). In our study, the conidia, germinated conidia and hyphae of all studied fungal species can be considered resistant to amphotericin B, because MIC values above 2 µg/mL have been associated with the treatment failure of aspergillosis (Lass-Florl et al. 1998). Correlation of minimal effective concentration concerning the clinical outcome to caspofungin must be yet elucidated; however, we do assume that the three morphotypes of all fungal species tested in our work were resistant to this antifungal drug, because the protocol published by CLSI document M38-A2 (CLSI 2008) recommends its use in a concentration range varying from 0.015-8 µg/mL and, in our records, the MICs were always equal or higher than 8 µg/mL. The same situation was observed for fluconazole, in which the concentration range recommended by CLSI is 0.125-64 µg/mL (CLSI 2008). Our results pointed out that hyphae of all fungal species were resistant to fluconazole (MICs ≥ 256 µg/mL) as well as the germinated conidia of both *L. prolificans* and *S. apiospermum* (MIC > 256 µg/mL) and conidia of *L. prolificans* (MIC > 256 µg/mL), while the remaining species and conidia of *S. apiospermum* had a MIC lower than 16 µg/mL, and fungal strains with MICs lower than 64 µg/mL are considered susceptible to this drug (Saracli et al. 2003). Hyphae of all species as well as conidia and germinated conidia of *L. prolificans* and *S. apiospermum* were also considered resistant (> 8 µg/mL) to itraconazole, whereas both conidial and germinated conidial cells of *S. minutisporum, S. aurantiacum* were susceptible to this drug. Voriconazol was the most effective drug against conidia and germinated conidia of *S. apiospermum*, *S. aurantiacum*, *S. minutisporum* and *L. prolificans*, which is in agreement with data found by other authors in the literature (Lackner et al. 2012, Biswas et al. 2013); however, hyphae of *S. minutisporum* (MIC 32 µg/mL) and *S. aurantiacum* (MIC 128 µg/mL) presented higher MIC than the maximum concentration (16 µg/mL) for this antifungal proposed by CLSI (2008). However, voriconazole has multiple drug interactions with medications used in immunosuppression of organ transplant recipients, which does not allow its use in those cases (O’Bryan 2005). Thereby, the optimal treatment to combat the infections caused by *Scedosporium/Pseudallescheria* and *Lomentospora* in immunosuppressed patients is still completely unknown (Lackner et al. 2012).

Concisely, our data are in concordance with Lackner et al. (2012), who showed that *Scedosporium* species do not have a normal MIC distribution, which generates a great difficulty to select a drug to be used in clinical settings. These findings point out to the necessity to the accurate differential diagnosis of these fungal species in order to permit a correct clinical treatment.

Conidial germination is a crucial developmental stage in the life cycle of all filamentous fungi, since the outgrowth of conidia plays significant roles in their dispersal as well as in several steps of the interaction with key host structures. In fact, the morpho-biochemical transition from dormant conidia into active, growing, filamentous hyphae requires the coordination of numerous biosynthetic, developmental and metabolic processes. Taken together, our data demonstrate that *S. apiospermum*, *S. aurantiacum*, *S. minutisporum* and *L. prolificans* conidial cells differentiate into an invasive form under diverse concentration of nutrients, pHs and temperatures, which can reflect their abilities to colonise several sites of human body and natural environments. CO_2_ was a substantial inducer of the conidia-into-hyphae transformation in *S. apiospermum*, *S. aurantiacum*, *S. minutisporum* and *L. prolificans*. Furthermore, the susceptibility to antifungals was dependent of the cell morphotype, with hypha highly resistant to the majority of the tested drugs. In brief, we can conclude that our results add novel data to clarify the complex phenomenon regarding the crucial transition of conidia into filamentous form in these fungal species. Finally, studies on the cell differentiation mechanisms may also aid the elucidation of antifungal resistance of these relevant human opportunistic pathogens.
